# Fragile X-associated tremor ataxia syndrome with co-occurrent progressive supranuclear palsy-like neuropathology

**DOI:** 10.1186/s40478-019-0818-z

**Published:** 2019-10-30

**Authors:** Amanda N. Sacino, Stefan Prokop, Meggen A. Walsh, Jennifer Adamson, S. H. Subramony, Amy Krans, Peter K. Todd, Benoit I. Giasson, Anthony T. Yachnis

**Affiliations:** 1 Department of Neuroscience, College of Medicine, University of Florida, Gainesville, FL USA; 2 Center for Translational Research in Neurodegenerative Disease, University of Florida, Gainesville, FL USA; 3 Present address: Department of Neurosurgery, Johns Hopkins Hospital, Baltimore, MD USA; 4 Department of Pathology, College of Medicine, University of Florida, Gainesville, FL USA; 5 Norman Fixel Institute for Neurological Diseases, University of Florida, Gainesville, FL USA; 6 Department of Neurology, College of Medicine, University of Florida, Gainesville, USA; 7 VA Ann Arbor Healthcare Center, Ann Arbor, MI USA; 8 Department of Neurology, University of Michigan, Ann Arbor, MI USA

**Keywords:** FXTAS, PSP, RAN-translation

## Abstract

Co-occurrence of multiple neuropathologic changes is a common phenomenon, most prominently seen in Alzheimer’s disease (AD) and Parkinson’s disease (PD), complicating clinical diagnosis and patient management. Reports of co-occurring pathological processes are emerging in the group of genetically defined repeat-associated non-AUG (RAN)-translation related diseases. Here we report a case of Fragile X-associated tremor-ataxia syndrome (FXTAS) with widespread and abundant nuclear inclusions of the RAN-translation related FMRpolyG-peptide. In addition, we describe prominent neuronal and glial tau pathology representing changes seen in progressive supranuclear palsy (PSP). The highest abundance of the respective pathological changes was seen in distinct brain regions indicating an incidental, rather than causal correlation.

## Introduction

Fragile X-associated tremor-ataxia syndrome (FXTAS) was first discovered in 2001 and has emerged as a unique late onset ataxia with often characteristic imaging features [9, 11, 17]. FXTAS is caused by a premutation expansion in the trinucleotide CGG repeat (50–200 repeats) in the fragile X mental retardation 1 (*FMR1*) gene. Patients typically present from 60 to 65 years of age with tremor and/or gait ataxia and a spectrum of associated neurologic and medical symptoms [9]. Diagnostic criteria also include pathognomonic neuroimaging and neuropathological findings [1, 8, 10]. The intranuclear sequestration of proteins by expanded CGG repeats in FMR1 mRNA has been suggested as a triggering pathogenic event [7]. In addition, non-AUG-initiated (RAN) translation of the expanded CGG repeats into a polyglycine-containing peptide (FMRpolyG) that is prone to aggregate has been implicated in neurotoxicity [2, 3, 15, 18]. These aberrant protein inclusions can also trigger the aggregation of other proteins. Indeed, there have been reports of the co-occurrence of FXTAS with Lewy body dementia (LBD) and Alzheimer’s disease (AD) [16]. We now report a patient with FXTAS and neuropathological evidence of co-occurring progressive supranuclear palsy (PSP).

## Case presentation

A 65-year-old Caucasian male was followed in the ataxia clinic at the Center for Movement Disorders and Neuro-restoration at the University of Florida who had a 10-year history of gradually progressive bilateral upper extremity postural tremor and action myoclonus, bradykinesia, memory impairment, and an unsteady gait with frequent forward falls. On exam he had no vision abnormalities or gaze palsies and normal muscle strength with intact sensation, but limited cervical rotation due to neck muscle stiffness. MRI revealed FLAIR and T2 hyperintensities in the middle cerebellar peduncles extending into the deep white matter of the cerebellum as well as generalized atrophy. Given that the patient’s clinical presentation and imaging were suggestive of FXTAS, further genetic testing was completed which showed 96 CGG trinucleotide repeats in the *FMR1* gene. By age 68, he had diffuse myoclonus and a severely ataxic gait requiring the use of a wheelchair, inability to perform activities-of-daily-living due to impairment of executive functioning, dysarthria, dysmetria, hypomimia, incontinence, and constipation. He died at the age of 69.

Central nervous system autopsy was performed with post-mortem interval of 3 h. The brain weighed 1235 g and gross examination revealed gray discoloration of the cerebellar peduncles and deep cerebellar white matter. There was mild hypopigmentation of the substantia nigra.

**Fig. 1:**
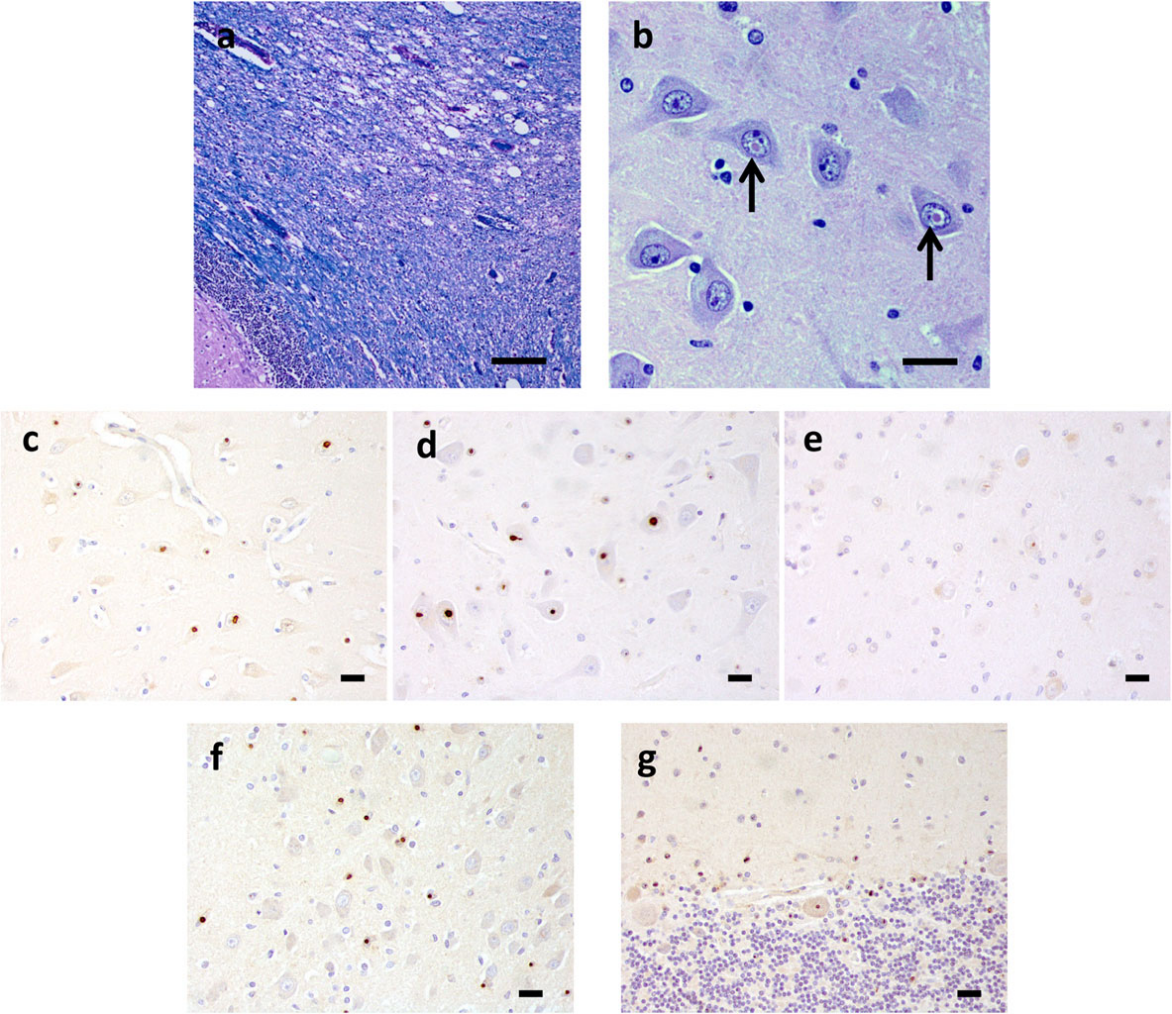
Neuropathology of FXTAS. Luxol fast blue-H&E stain shows spongiosis of the cerebellar white matter (**a**). Abundant eosinophilic intranuclear inclusions (arrows) were found in neurons of the cerebral cortex, especially in hippocampal pyramidal cells (**b**). Intranuclear inclusions in the CA4 region of the hippocampus were immunorective for p62 (**c**), NTF1 (**d**) and CTF1 (**e**). Neurons and glia in pontine nuclei (**f** NTF1), as well as Bergmann glia and rare Purkinje neurons of the cerebellum also contained intranuclear inclusions (**g** NTF1). [Scale bar = 100 μm in **a**; 20 μm in **b**, **c**, **d**, **e**, **f** and **g**]

Routine H&E and Luxol-fast blue-H&E stains were examined and immunohistochemical studies for tau (PHF-1, Peter Davies, 1:500; AT-8, Fisher 1:250; RD4, Millipore, 1:1000), α-synuclein (pSer 129, 81A [19] 1:5000), Aβ (33.1.1; 1:1000), TDP-43 (pSer409/410; Proteintech 1:1000), ubiquitin (Abcam, 1:500), p62 (Proteintech 1:250), GFAP (Promega, 1:1000), and RAN translation product specific antibodies NTF1 ([12]; 1:400) and CTF1 ([12]; 1:40) were performed. There was prominent spongiosis in the deep cerebellar white matter and middle cerebellar peduncles (Fig. 1a). Spongiosis was also present in the centrum semiovale and subcortical white matter of the cingulate gyrus. Abundant eosinophilic intranuclear inclusions were identified by routine H&E staining (Fig. 1b, arrows). These inclusions were immunoreactive for ubiquitin, p62 (Fig. 1c), NTF1 (Fig. 1d), a polyclonal antibody raised against the N-terminus of the FMRpolyG RAN translation product and focally also with CTF1 (Fig. 1e), a polyclonal antibody raised against the C-terminus of the FMRpolyG RAN translation product ([12] and accompanying manuscript ANEC-D-19-00289). These aggregates were found within neurons and protoplasmic astrocytes of the cerebral cortex, brainstem, cerebellum, and cervical spinal cord. Intranuclear inclusions were especially numerous in hippocampal dentate neurons, pyramidal neurons of CA3 and CA4 (Fig. 1 c, d, e), pontine nuclei (Fig. 1f) and frontal neocortical neurons. Foci of the cerebellar cortex showed Purkinje cell loss and intranuclear inclusions of Bergmann glia as well as rare Purkinje neurons (Fig. 1g).

**Fig. 2:**
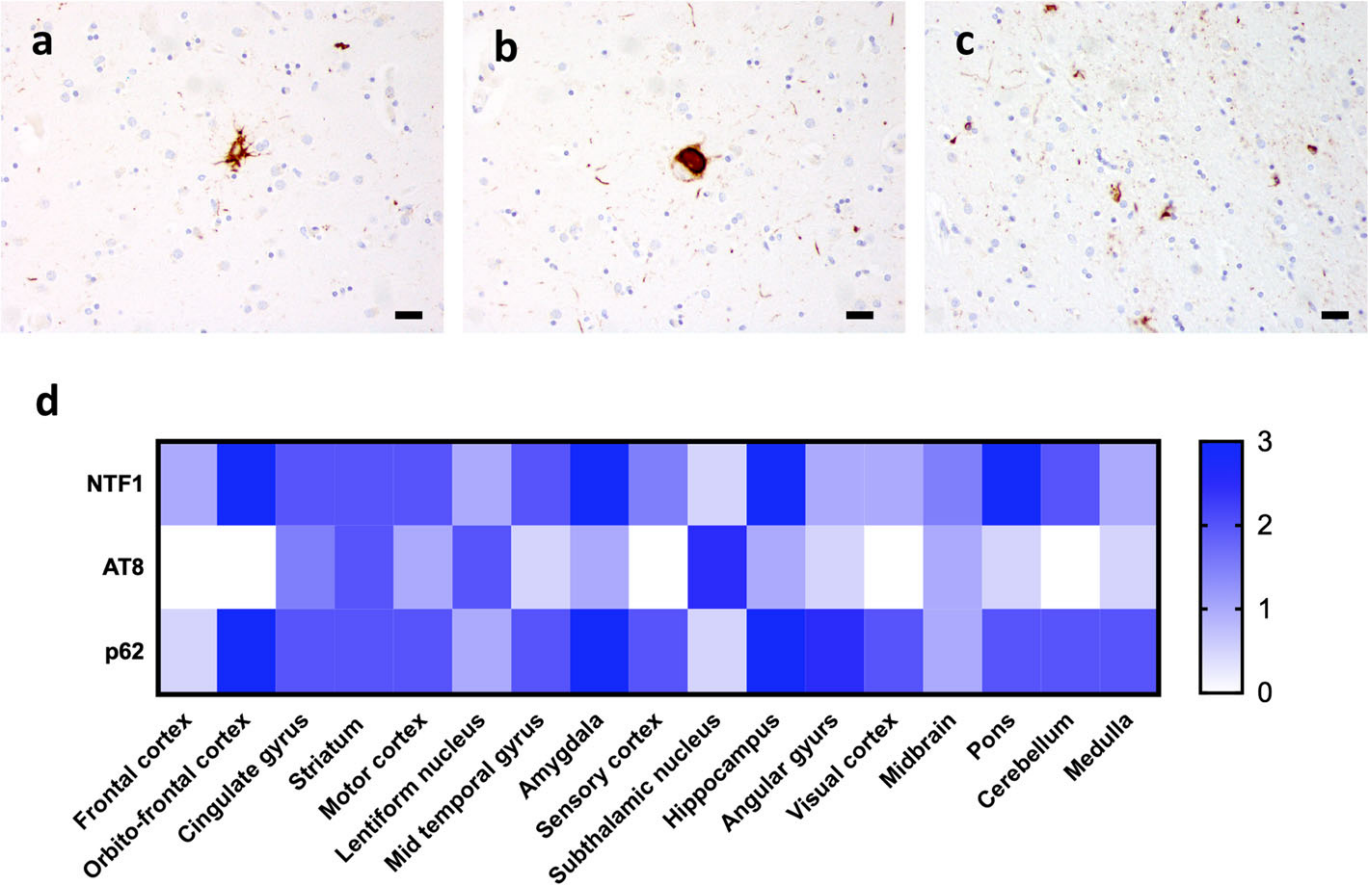
PSP-like changes were detected by AT8 immunohistochemistry as tufted astrocytes (**a**), globose tangles (**b**) and coiled bodies (**c**). (**d**) Heatmap of relative abundance of NTF1-positive pathology (upper row), AT-8 positive pathology (middle row) and p62-positive pathology (lower row). 0 = no pathology, 1 = mild pathology, 2 = moderate pathology and 3 = severe pathology). Scale bar: 20 μm

An immunocytochemical study for tau with AT8 and RD4 (4-repeat tau) antibodies demonstrated tufted astrocytes (Fig. 2a), globose neurofibrillary tangles (Fig. 2b) and oligodendroglial “coiled bodies” (Fig. 2c) in the basal ganglia, subthalamic nucleus, substantia nigra, amygdala, and medulla. In the substantia nigra and the locus coeruleus, tau pathology was associated with mild to moderate neuronal loss. No Aβ-amyloid, α-synuclein, or TDP-43 pathology was observed by immunohistochemical study. Plotting the relative abundance (0 = no pathology; 1 = mild pathology; 2 = moderate pathology and 3 = severe pathology) of intranuclear pathology (NTF1 and p62 staining) and tau pathology (AT8 staining), we noted a remarkable correlation between NTF1 staining and p62 staining (Fig. 2d, [12]). In contrast, areas with the most abundant tau pathology (lentiform nucleus and subthalamic nucleus) showed only minimal intranuclear pathology and vice versa (Fig. 2d).

## Discussion and conclusions

Patients with FXTAS may occasionally present with PSP-like clinical findings [5], and a recent case report showed co-occurrence of FXTAS with PSP neuropathological findings [14]. We have expanded upon these findings and report a case of abundant FXTAS pathology with co-occurring PSP-like tau pathology. Clinically, the patient had symptoms of FXTAS with tremor and ataxia with the addition of PSP-like symptoms of bradykinesia, postural instability, dysarthria, and executive dysfunction. However, there was no evidence of truncal rigidity or supranuclear gaze palsy [6]. Neuropathological study revealed classic changes of FXTAS including white matter pallor and spongiosis in the cerebellum and cerebrum along with widespread intranuclear ubiquitin, p62, and FMRpolyG positive inclusions [7, 8, 12]. Additionally, the patient had tau pathology including globose NFTs in the substantia nigra, locus coeruleus, medulla, and basal ganglia with tufted astrocytes and oligodendroglial coiled bodies composed of 4-repeat tau, which are consistent with PSP neuropathology, and given the overall low abundance possibly reflecting “incidental PSP” [4]. Cross-seeding of misfolded proteins has been suggested as a possible mechanism for the co-occurrence of multiple pathologies in neurodegenerative diseases [13], but whether our case reflects the incidental co-occurrence of two distinct neurodegenerative processes or is the consequence of a cross-talk between two distinct neurodegenerative processes cannot be determined with certainty. The fact that the highest abundance of the respective neuropathological changes was detected in non-overlapping brain regions may favor two independent processes.

## Data Availability

All data generated or analysed during this study are included in this published article.

## Abbreviations

AD: Alzheimer’s disease
FXTAS: Fragile X-associated tremor-ataxia syndrome
LBD: Lewy body dementia
NFT: Neurofibrillary tangle
PD: Parkinson’s disease
PSP: progressive supranuclear palsy
RAN: Repeat-associated non-AUG
